# A Novel Method for Drug Screen to Regulate G Protein-Coupled Receptors in the Metabolic Network of Alzheimer's Disease

**DOI:** 10.1155/2018/5486403

**Published:** 2018-02-20

**Authors:** Yang Li, Wei Zheng, Wuyun Qiqige, Shujuan Cao, Jishou Ruan, Yanping Zhang

**Affiliations:** ^1^College of Life Sciences, Nankai University, No. 94 Weijin Road, Tianjin 300071, China; ^2^College of Mathematical Sciences and LPMC, Nankai University, No. 94 Weijin Road, Tianjin 300071, China; ^3^Department of Computational Medicine and Bioinformatics, University of Michigan, Ann Arbor, MI 48109, USA; ^4^Computer Science and Engineering Department, Michigan State University, East Lansing, MI 48823, USA; ^5^School of Science, Tianjin Polytechnic University, No. 399 Binshui West Road, Xi Qing District, Tianjin 300387, China; ^6^State Key Laboratory of Medicinal Chemical Biology, Nankai University, No. 94 Weijin Road, Tianjin 300071, China; ^7^School of Mathematics and Physics, Hebei University of Engineering, No. 199 South Street Light, Handan 056038, China

## Abstract

Alzheimer's disease (AD) is a chronic and progressive neurodegenerative disorder and the pathogenesis of AD is poorly understood. G protein-coupled receptors (GPCRs) are involved in numerous key AD pathways and play a key role in the pathology of AD. To fully understand the pathogenesis of AD and design novel drug therapeutics, analyzing the connection between AD and GPCRs is of great importance. In this paper, we firstly build and analyze the AD-related pathway by consulting the KEGG pathway of AD and a mass of literature and collect 25 AD-related GPCRs for drug discovery. Then the ILbind and AutoDock Vina tools are integrated to find out potential drugs related to AD. According to the analysis of DUD-E dataset, we select five drugs, that is, Acarbose (ACR), Carvedilol (CVD), Digoxin (DGX), NADH (NAI), and Telmisartan (TLS), by sorting the ILbind scores (≥0.73). Then depending on their AutoDock Vina scores and pocket position information, the binding patterns of these five drugs are obtained. We analyze the regulation function of GPCRs in the metabolic network of AD based on the drug screen results, which may be helpful for the study of the off-target effect and the side effect of drugs.

## 1. Background

Alzheimer's disease (AD) is a chronic and progressive neurodegenerative disorder. The pathogenesis of AD is poorly understood [1]. There is as yet no safe and effective drug treatment for stopping or reversing the progression of AD. There exist three main hypotheses for the pathogenesis of the disease: (i) cholinergic hypothesis which maintains that decreased acetylcholine (ACh) contributes to the cognitive decline commonly observed in AD [2]; (ii) amyloid hypothesis which supposes that the accumulation of amyloid-*β* (A*β*) is the fundamental cause of AD [3]; (iii) tau protein hypothesis which holds that the dysfunction of hyperphosphorylated microtubule-associated protein tau results in neurofibrillary lesions and then initiates AD [4].

G protein-coupled receptors (GPCRs) constitute a large receptor family that possess seven transmembrane helices [5] and play a key role in the pathogenesis of AD. Acquiring the crystal structure of a GPCR is the first step in analyzing the metabolic mechanism of GPCR in AD and further researching the binding site information related to AD. However, experimental structure determination remains difficult for GPCRs [6]. Fortunately, there are some protein 3D structure prediction methods developed for this problem, such as I-TASSER [7], Rosetta [8], and Quark [9]. Therefore, we employ the GPCR structure models predicted by GPCR-I-TASSER pipeline, which is a computational method designed for 3D structure prediction of G protein-coupled receptors, to analyze the binding site information.

**Figure 1:**
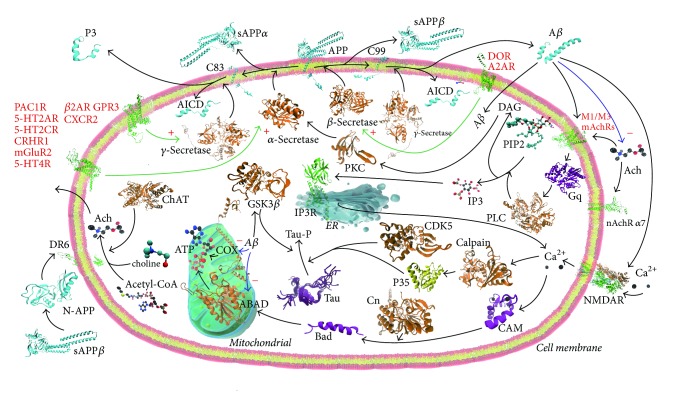
Metabolic pathway of A*β* and GPCRs in Alzheimer's disease. The crystal structure of each protein is shown in a secondary structure cartoon representation, whereas each ligand is showed in a ball-and-stick model. The APP related proteins are painted in blue. The receptors are painted in green while the ion channel receptor (NMDAR) is painted in orange and green. The enzymes are painted in orange while the activator of an enzyme (p35) is painted in yellow. And other proteins are painted in purple. The GPCRs are marked with red descriptive texts. Black arrows represent direct molecular interaction while blue arrows with a minus sign represent the inhibiting effect. Green arrows with a plus sign represent the agonist stimulation of the GPCRs activating the related enzymes.

To fully understand the pathogenesis of AD and design novel drug therapeutics, analyzing the AD pathway is of great importance.  Figure 1 shows the AD-related pathway which is built by consulting the Kyoto Encyclopedia of Genes and Genomes (KEGG) pathway of AD and a mass of literatures [10–13]. Amyloid-*β* (A*β*) deposition can be affected by the cleavage of amyloid precursor protein (APP). APP has two processing pathways. Firstly, the *α*-secretase catalyzes the hydrolysis of APP to the soluble N-terminal ectodomain of APP (sAPP*α*) and the C83. Subsequently, the *γ*-secretase catalyzes the hydrolysis of C83 to the APP intracellular C-terminal domain (AICD) and P3. Secondly, the *β*-secretase catalyzes the hydrolysis of APP to the sAPP*β* and C99. Subsequently, the sAPP*β* is cleaved into N-APP which binds death receptor 6 (DR6, also known as TNFRSF21), leading to neuronal and axonal degeneration, the main markers of AD [14]. And the further cleavage of C99 generates the AICD and A*β* by the *γ*-secretase. In the above APP processing, GPCRs play a key role in modulating the pathway. The *β*_2_ adrenergic receptor (*β*_2_AR), G protein-coupled receptor 3 (GPR3), and CXC-chemokine receptor 2 (CXCR2) have effect on modulating the cleavage of C99 or C83 by *γ*-secretase. For example, the administration of a *β*_2_AR antagonist promotes the *γ*-secretase-mediated cleavage of C99 or C83 and then reduces the amyloid plaque burden [11]. The *δ*-opioid receptor (DOR) and the adenosine A2A receptor (A_2A_R) have effect on modulating the cleavage of APP by *β*-secretase. For example, the treatment with a DOR antagonist promotes the cleavage of APP by *β*-secretase and then decreases the A*β* deposition [15]. Similarly, the cleavage of APP by *α*-secretase can be modulated by corticotrophin-releasing hormone (CRH) receptor type I (CRHR1), 5-hydroxytryptamine (5-HT) receptor subtypes for 5-HT_2A_R, 5-HT_2C_R, and 5-HT_4_R, metabotropic glutamate receptor 2 (mGluR2), and pituitary adenylate cyclase 1 receptor (PAC1R). After binding a ligand, the GPCR like muscarinic acetylcholine receptor M3/M1 (M3/M1 mAchRs) is coupled to a Gq protein resulting in the activation of PLC which catalyzes the hydrolysis of phosphatidylinositol 4,5-bisphosphate (PIP2) into inositol triphosphate (IP3) and diacylglycerol (DAG). Subsequently, IP3 binds to IP3 receptors (IP3R) on the endoplasmic reticulum [16], causing the release of calcium (Ca^2+^), whereas DAG remaining to plasma membrane can activate protein kinase C (PKC) which results in the activation of *α*-secretase and then decreases the A*β* generation [11]. M3/M1 mAchRs are the receptors of A*β* and acetylcholine (Ach) which is synthesized by the transfer of an acetyl group from acetyl-CoA to choline catalyzed by choline acetyltransferase (ChAT). A*β* and Ach are two crucial influences in AD. The release of Ach can be reduced by A*β* deposition [11]. Ach has many other receptors such as nicotinic acetylcholine receptor subunit alpha-7 (nAchR *α*7), whereas A*β* binds to the N-methyl-D-aspartate receptor (NMDAR) which is an ion channel receptor [11]. Extracellular calcium can pass through the cell membranes by NMDAR. Then, intracellular calcium binds to calpain which catalyzes the cleavage of cyclin-dependent kinase 5 activator 1 (p35) into a p25 form. p35 is an activator of cyclin-dependent-like kinase 5 (CDK5), and they together can phosphorylate the tau protein [17]. The enzyme glycogen synthase kinase 3*β* (GSK3*β*) can also catalyze tau phosphorylation [18].

## 2. Methods and Materials

### 2.1. Docking Methods

#### 2.1.1. AutoDock Vina

The molecular docking is a computational strategy commonly hired in drug discovery to conduct virtual screen (VS) before any costly and time-consuming biologic assays [20]. AutoDock Vina is one of the most popular multi-CPU docking program with enhanced speed of execution and accuracy of binding mode prediction [21]. AutoDock Vina can not only predict the conformation of protein-ligand complex but also provide the assessment of binding affinity. Therefore, promising drug-like ligands can be enriched by docking with target proteins at an atom level and the interaction of protein-ligand also can be modeled for further exploration [22].

#### 2.1.2. ILbind

ILbind is a consensus-based approach that aims to provide improved predictive quality [23]. It is derived from two methods, FINDSITE [24] and SMAP [25], which are developed to predict binding pockets for specific drug ligand. Depending on structural information of a few protein-ligand complexes from experiment or database, the prediction routines can help to find secondary therapeutic and off-targets of a given drug molecule on a proteomic scale. Combining with these two approaches by using an ensemble of Support Vector Machines (SVMs), ILbind is shown to offer effective prediction of protein-ligand binding, even though the targets protein is structurally distant from known complexes and successfully used in hunting for off-targets interaction [26].

### 2.2. Dataset

#### 2.2.1. GPCR Proteins

We collect 25 G protein-coupled receptors involved in the metabolism of Alzheimer's disease, which are listed in Table S1, based on the AD-related pathway (Figure 1) and some related literature [10–13]. For the reason that many GPCRs do not have complete crystal structure, we construct the 3D structure models of all 25 GPCRs either by searching in the GPCR-HGmod [6] database or implementing the GPCR-I-TASSER tool. GPCR-I-TASSER [7] is an online web server designed for high-accuracy 3D structure prediction of G protein-coupled receptors. The server is developed by Zhang' Lab, University of Michigan. GPCR-HGmod is a database which contains 3D structure models of human GPCRs, and its models are all generated by GPCR-I-TASSER pipeline. First, all GPCR models are downloaded from GPCR-HGmod database. But for those incomplete models or multidomain models, we manually generate them by GPCR-I-TASSER. For each GPCR, five models can be generated based on either GPCR-HGmod database or GPCR-I-TASSER pipeline. The model with the smallest Root Mean Square Deviation (RMSD) against experimental fragments of crystal structure is selected for next docking step. For GPCRs that do not have any experimental structural fragments, Uniprot topology knowledge is used for selecting model [27]. Finally, 22 GPCR models are selected in this paper (see detailed information in Table S1).

#### 2.2.2. Drugs for GPCR

Drugs used to screen against the GPCR targets are selected depending on the Anatomical Therapeutic Chemical Classification System (ATC) [28]. The goal of this research is to find GPCR-related off-target drugs or potential drugs that may intervene with the pathway of the disease. According to corresponding organ or system of AD, 33 drugs are assembled by their ATC code (Table S2), including 26 approval drugs, 3 experimental drugs, 3 nutraceutical drugs, and 1 withdrawal drug. These drugs mainly aim at (1) alimentary tract and metabolism (ATC code “A”), (2) blood and blood forming organs (ATC code “B”), (3) cardiovascular system (ATC code “C”), and (4) nervous system (ATC code “N”).

A cross-validation method is conducted on the GPCR dataset with 22 GPCR proteins. That is, we enumerate all the pairs of selected drugs and GPCRs in the dataset and each pair is evaluated to determine if they could interact and combine into complex. Finally, 726 pairs are generated and checked in this study.

We scan Uniprot database [27] and find only 11 experimental structures of GPCR targets can form complex structure with some drugs (4 drugs included in our selected 33 drugs) because experimental structure determination for GPCRs remains a difficult problem. And only one of these 11 therapeutic targets (beta-2 adrenergic receptor) is also included in our 22 CPCRs. Therefore, it is impossible to infer the binding information between the 22 AD-related GPCRs and 33 selected drugs only based on these limited data and information from experimental crystal GPCR structure. Alternatively, we build the DUD-E dataset in this study as described below.

#### 2.2.3. DUD-E Dataset

To quantitatively assess the performance of the hybrid method combining ILbind and AutoDock Vina and provide a baseline for the detection of AD-related drugs, we choose the Directory of Useful Decoys, Enhanced (DUD-E) dataset, as the train dataset [29].

Some datasets have been developed in the previous study of drug screen [30]. Two basic molecules, of which the capacity of combining with target proteins has been confirmed by experiment or previous study, are collected: ligand (refers to molecules that can form complexes with target proteins) and decoy (refers to molecules that cannot form complexes with target proteins). However, without considering the similarity of physical properties between ligand and drug-like decoy, this dataset is biased on screen evaluation. For example, molecule weight has significant influence on the docking score, which leads to artificial good performance on distinguishing ligand and decoy if they have very different molecule weights [31].

DUD-E tackles this weakness by matching the decoy drug according to the physical chemistry of ligand molecule [32]. Six physical properties, molecular weight, number of rotatable bonds, hydrogen bond donors, hydrogen acceptors, LogP, and net charge, are considered when assembling the sets of ligands and decoys.

For ILbind-based drug screen, the templates of each ligand are prerequisite. We compare the DUD-E dataset with two databases, DrugBank [33] and PDB (Protein Data Bank) [34], to pick up targets that fulfill the requirement. First of all, targets in DUD-E are assigned at least one drug in DrugBank as the ligand to pick up. Secondly, the decoys of these targets are mapped to PDB chemical component ID (HET ID). Finally, drug ligands and decoys without complex templates in PDB are filtered out.

At the end, 336 ligand/decoy samples are selected from DUD-E, which consist of 90 ligands and 246 decoys. The 336 samples can form complex structures with 43 targets including nuclear, kinases, proteases, GPCRs, and other enzymes. Therefore, a total of 336 target-ligand/decoy pairs are involved in the DUD-E dataset (Table S3).

#### 2.2.4. Estimation of Binding Baseline

To predict the potential combination between target proteins and drug molecules/ligand, an accurate, reliable, and computationally efficient screen method is needed. Here, we adapt a recently developed inverse ligand binding predictor, ILbind. Meanwhile, the widely used molecular docking program, AutoDock Vina, is also employed.

The prediction of protein-ligand binding by ILbind is mainly determined by both segment alignment and profile-profile alignment between target proteins and the known protein-drug complex templates. As a consequence, this approach suffers the defect that it may filter out the direct structural information, such as pocket cavity and binding residues. To characterize the protein-ligand interaction and examine the off-target screen in a structural-based way, AutoDock Vina is performed on each protein-ligand pair as the complementary of more position specific details. All the parameters of docking procedure by AutoDock Vina are kept as their default value. And as the binding pocket on the receptor is unknown, we perform the docking in a “blind” way. That is, the grid box used in docking fully contains the whole receptor as its search space.

In a cycle of virtual screen, a disease-centered interaction network (Figure 1) is first constructed and GPCR target proteins are collected and built the 3D structures base on GPCR-HGmod or GPCR-I-TASSER. Then drugs of each target protein are collected from DrugBank according to the ATC category. The GPCR-drug pairs for drug screen are generated by enumerating the prepared drugs and GPCR target proteins to ensure the coverage of the possible complex combination in the network. The DUD-E dataset is used to train the baseline or cutoff of ILbind binding score for picking up GPCR-drug pairs. Then the AutoDock Vina is utilized to improve the precision of the screen and obtain the GPCR-drug combination pattern in a structural level.

#### 2.2.5. Performance Evaluation Metrics

To evaluate the performance of the hybrid method combining ILbind and AutoDock Vina, accuracy (ACC), sensitivity (TPR or recall), and AUC are used in this paper.

(1) ACC=TP+TNTP+TN+FP+FN,TPR=TPTP+FN,

where TP and TN are the number of correctly predicted binding pairs and nonbinding pairs, respectively, FP is the number of nonbinding pairs that were predicted to be binding pairs, and FN is the number of binding pairs that were predicted not to be binding pairs.

## 3. Results and Discussion

### 3.1. Construction of GPCR 3D Structures

From the AD-related pathway (Figure 1) and some literature [10], 25 GPCRs are reported referring in AD pathway. Most of them do not have complete crystal structure. We use GPCR-HGmod or GPCR-I-TASSER to build 22 structure models. Details for 22 models are listed in Table S1. For each GPCR, five models can be generated based on GPCR-HGmod or GPCR-I-TASSER. Our selection criterion is based on the smallest Root Mean Square Deviation (RMSD) against experimental fragments of crystal structure. For example, corticotropin-releasing factor receptor 1 (CRHR1), which is reported to infer to mediate the level of sAPP*α* through *α*- secretase, is a 444 amino acids protein. There are two different domain structural fragments in PDB. 3EHU is extracellular domain from 24th residue to 119th residue in sequence. Model 1 of CRHR1 (see Figure S1) in GPCR-HGmod database has the smallest RMSD (1.16 Å) with 3EHU. 4K5Y consisting of 7 transmembrane helixes is experimental crystal structure of CRHR1 from 104th residue to 373rd residue. The RMSD value of model 1 with 4K5Y is 0.66 Å. Therefore, model 1 of CRHR1 in GPCR-HGmod database is our best choice in this paper, and then this model will be used in the ligand docking stage. Other models of GPCRs (e.g., M3 mAchR, DOR, A_2A_R) are selected adopting the same selection criterion as CRHR1. However, there are some GPCRs that do not have any experimental structural fragments. Thus, Uniprot topology knowledge is used for selecting model. For example, AT_2_R is a receptor which has 363 amino acids. References have implicated that AT_2_R may take part in several CNS (Central Nervous System) functions, including neuronal apoptosis, behavior, and memory [11]. There is not any crystal structure of AT_2_R in PDB database. So Uniprot topology information and predicted second structure information will be used to select the best model in GPCR-HGmod models. The extracellular domain is located from 1st residue to 45th residue which is predicted as coil by Psipred [35] and SpineX [36–38], while the extracellular domain of model 1 in GPCR-HGmod is constructed as helix. Therefore, model 1 of AT_2_R is not a good choice. Finally, model 2 of AT_2_R in GPCR-HGmod has been selected as a 3D structure model in this paper. Other GPCRs (e.g., M1 mAchR, GPR3, 5-HT_6_R) are selected using the similar criterion. There is no model selected for mGluR1, mGluR5, and GLP1R. For example, 3IOL is a fragment crystal structure of GLP1R extracellular domain. All five models in GPCR-HGmod have bad RMSD value with 3IOL. As a result, there is no model selected for GLP1R in this paper. Thus, we construct 22 GPCR models related to AD pathway.

### 3.2. Estimation of Baseline for ILbind Score Based on DUD-E Dataset

As the previous description, we perform ILbind-based screen and AutoDock Vina-based screen on each pair of ligand and target in DUD-E dataset listed in Table S3, respectively. ILbind scores imply the probability of interaction between ligand and target protein. Meanwhile, AutoDock Vina scores represent the predicted affinity of ligand-target complex and give the combination pocket pattern. As AutoDock Vina docking is configured to search the global grid box that the target protein is totally contained in, the conformation of predicated complex corresponding to the lowest affinity score is considered as the most robust one.

**Figure 2:**
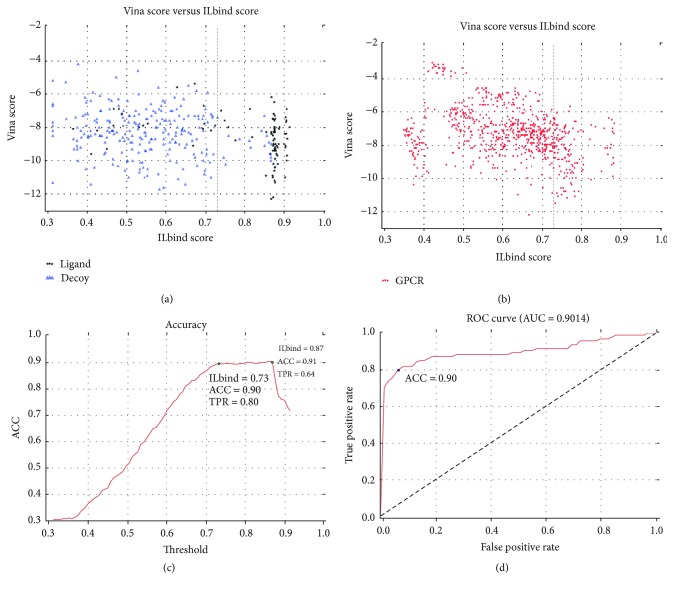
(a) Distribution of ILbind binding score and AutoDock Vina affinity based on DUD-E dataset. *X*-axis represents ILbind score and *y*-axis is minimal AutoDock Vina affinity. Protein and decoy pairs are shown as blue points, while target and ligand pairs are shown as black points (the detailed descriptions are in Table S3). (b) The cross-testing results between 22 GPCRs and 33 drugs (the detailed descriptions are in Tables S1 and S2). Dashed line means the cutoff 0.73 of ILbind binding score. (c) ACC curve of ILbind classifier based on different thresholds. (d) ROC curve of ILbind classifier. AUC is 0.90, ACC is 0.90, and TPR is 0.80 with the threshold 0.73.

The distribution of both ILbind score and AutoDock Vina score based on DUD-E dataset, which includes 336 target-ligand/decoy pairs (Table S3), is plotted in Figure 2(a). It is obvious that the target-ligand/decoy pairs can by clustered by their ILbind scores and most target-ligand pairs are located between 0.7 and 1.0. However, several target-decoy pairs are still presented in this range. The proportion of target-decoy pairs increases rapidly as the ILbind score falls below 0.7.

To predict the GPCR-drug pairs that are most likely to interact and form complexes, we assess and estimate the baseline of docking score of ILbind and AutoDock Vina based on the DUD-E dataset in a two-stage way.

Firstly, the ACC of ILbind classifier is evaluated based on different ILbind scores in order to provide a visible gap that draws a line between the ligands and decoys (Figure 2(c)). Both ACC and sensitivity are considered to determine the baseline or cutoff of ILbind score used in drug screen (Figure 2(c)). The max ACC score (0.91) is achieved by setting ILbind score to 0.87. And we can find the ACC score merely changes 0.01 when the ILbind score is increased from 0.73 to 0.87. However, the sensitivity falls down from 0.80 to 0.64. As a tradeoff, ILbind score = 0.73 is selected as the edge between two categories (ligand and decoy). The result that AUC score is 0.9014 when ILbind score is selected as 0.73 demonstrates that ILbind is an efficient procedure to classify the ligand and decoy (Figure 2(d)).  Figure 2(b) shows the cross-testing results between 22 GPCRs and 33 selected drugs when the baseline of ILbind score is selected as 0.73.

Secondly, the AutoDock Vina scores are considered as an auxiliary factor when we exam the ligand-protein combination pattern in a structural level. Unlike the ILbind scores, the AutoDock Vina scores are spread out and the relationship between the scores and the classification of samples in DUD-E dataset is implicit. However, this feature is still helpful to facilitate the precision of the screen. The affinity score value smaller than −10.0 indicates a strong interaction between the ligand and target [21]. Meanwhile, AutoDock Vina predicts the conformation of complexes and provides the combination pattern information which can be analyzed at an atom level. As a consequence, a visualized check of the AutoDock Vina results is followed by the ILbind-based screen.

### 3.3. Drug Screen for GPCR Targets in AD Network

Drug screen for GPCR targets is conducted in a cross-testing way. By enumerating both the 33 selected drugs and 22 GPCR proteins with predicted structure, we got 726 drug-GPCR pairs (Figure 2(b)). According to the analysis of DUD-E dataset, we first screen these 726 pairs based on the baseline of ILbind scores (*⩾*0.73, selected based on DUD-E dataset). AutoDock Vina may give several scores for different binding patterns for a drug-GPCR pair. Therefore, we set the baseline of AutoDock Vina score to −9 kcal/mol (i.e., a drug-GPCR pair is selected, if there are any AutoDock Vina score of this drug-GPCR pair less than −9 kcal/mol). Since the binding patterns of the drug and GPCR should be located in extracellular domain or loop of GPCR, we further remove those pairs where no patterns with AutoDock Vina score ⩽ −9 kcal/mol are located in extracellular domain or loop of GPCRs. Finally, 45 drug-GPCR pairs are selected (Table S1), which includes 5 drugs, that is, Acarbose (ACR), Carvedilol (CVD), Digoxin (DGX), NADH (NAI), and Telmisartan (TLS). The analysis of the docking results is based on the four groups of GPCRs in the AD pathway (Figure 1).

**Figure 3:**
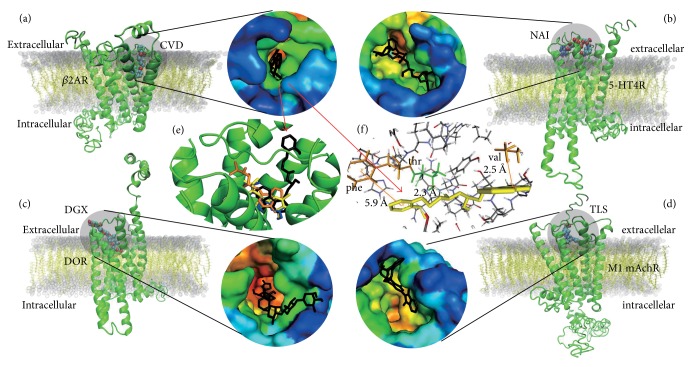
Binding information for GPCR-ligand pairs. Ligand is shown as sphere in subfigures (a), (b), (c), and (d), while surface pocket information of protein is shown in round in each subfigure. Protein surface is colored by the increasing of convex hull, which is calculated by CHOPS [19]. The color that shades from blue into red means convex hull layer increases from small to large. (a) CVD binds to extracellular domain of *β*_2_AR. (b) NAI binds to extracellular domain of 5-HT_4_R. (c) DGX binding pocket is in the extracellular domain of DOR. (d) TLS binds to the pocket of M1 mAchR extracellular domain. (e) Agonist Isoproterenol, clenbuterol, and antagonist ICI 118, 551 for *β*_2_AR also binds the same pocket with CVD. Isoproterenol is shown in blue color, clenbuterol is shown in yellow color, ICI 118,551 is shown in orange color, and CVD is shown by black color. (f) Binding interaction between *β*_2_AR and CVD (yellow). Terminal benzene ring of CVD forms *π*-*π* interaction with aromatic residue PHE194. On the opposite, VAL114 forms *σ*-*π* interaction with CVD. THR195 receives H^+^ to form hydrogen bond with CVD. *π*-*π* and *σ*-*π* interactions are shown by orange color and hydrogen bond is shown in green color.

*β* adrenergic receptor, consisting of *β*_1_AR and *β*_2_AR, is reported to be a therapeutic target of drug Carvedilol (CVD). Therefore, the pair of CVD and *β*_2_AR get a relatively high score in both ILbind screen (0.88) and AutoDock Vina docking (affinity = −9.9 kcal/mol). CVD binds to the extracellular domain of *β*_2_AR (Figure 3(a)) and forms *σ*-*π* interaction with residue VAL114 by its terminal benzene ring. On the opposite terminal, benzene binds to aromatic residue PHE194 to form a *π*-*π* bond with a distance of 5.9 Å. THR195 receives H+ to form hydrogen bond with CVD (Figure 3(f)). Among experimental crystal structures in PDB, we find *β*_2_AR complex with ligand CAU and *β*_1_AR complex with ligand CVD. The two X-ray structures suggest that CVD may bind to the same pocket for different experimental *β* adrenergic receptors (see Figure S2). *β*_2_AR can not only smooth heart muscle but also involve increasing or decreasing the level of A*β*_42_ and A*β*_40_ by mediating *γ*-secretase in brain. Studies implicate that A*β* can increase or decrease with the treatment of *β*_2_AR agonist or antagonist in AD transgenic mouse model. In our study, we find that CVD binds to the same pocket with *β*_2_AR agonist and antagonist (Figure 3(e)). It seems to be an evidence that CVD or CVD-like ligand will be a potential agonist or antagonist for *β*_2_AR in A*β* mediating pathway.

The 5-HT receptors (also known as serotonin receptors) include 7 subfamilies and 14 receptors. 5-HT_4_R is a kind of receptor that distributes in central nervous system, peripheral nervous system, and gastrointestinal tract. It has been reported to increase the level of sAPP*α* and decrease the level of A*β* by agonist prucalopride in a transgenic mouse model of AD. Docking result shows that DGX, NAI, and TLS can bind to the same pocket as prucalopride. 5-HT_4_R and NAI docking complex are shown in Figure 3(b). NAI forms two hydrogen bonds with residues GLU80 and ASP84, which are located in extracellular domain between TM2 and TM3. The ILbind score of this complex is 0.78 and affinity is −9.1 kcal/mol. This result is similar to the results of other family members of 5-HT receptors, such as 5-HT_2A_R, 5-HT_2C_R, and 5-HT_6_R.

There are two kinds of classical receptor for acetylcholine: nicotinic acetylcholine receptor (nAchR) which is a ligand-gated ion channel and muscarinic acetylcholine receptor (mAchR) which in contrast is not an ion channel but belongs to GPCR family. As reported by some studies, M1/M3 mAchR could modulate the level of A*β* through PLC pathway and activate PIP2 hydrolysis. Carbachol, cevimeline, atropine, and scopolamine are agonists for mAchR. We find TLS can bind to the extracellular domain of M1 mAchR which is the binding pocket for agonist carbachol (Figure 3(d)). Unlike M1 mAchR, M3 mAchR could bind CVD with a high ILbind score (0.88).

In contrast to 5-HT receptors or muscarinic acetylcholine receptors, DOR (*δ*-type opioid receptor) modulates the level of A*β*_40_ and A*β*_42_ by *β*- and *γ*-secretase. Agonist DADLE of DOR could lead to A*β* decreasing. Docking result has been implicated that DGX binds to the same pocket as DADLE with ILbind score 0.83, while TLS can form two *σ*-*π* interactions with DOR in this pocket (Figure 3(c)). Not similar to all GPCRs mentioned above, mGluR3 (metabotropic glutamate receptor 3) has a huge extracellular ligand binding domain, and there are plural pockets in this domain. DCG-IV is an agonist to mGluR3. AutoDock Vina docking result shows that binding positions of DCG-IV could be clustered to three pockets, and the largest cluster is located in the edge of extracellular domain to TM1. Ligands ACR and NAI could bind to this pocket. ACR especially is a high selective ligand for mGluR3 among 22 GPCRs selected in this paper.

**Figure 4:**
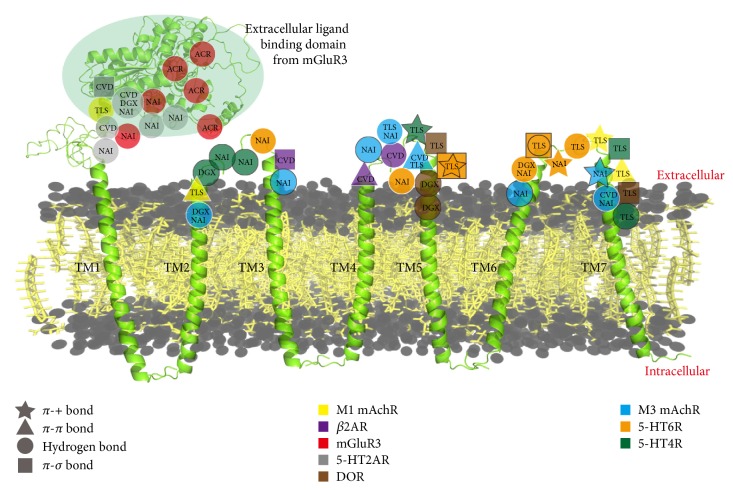
Binding distribution for GPCR-ligand pairs. The huge extracellular ligand binding domain is selected from mGluR3. Each shape means one kind of bond interactions. The residue with two shapes means this residue could form two kinds of bond interactions with the same ligand. For example, star mark means the residue can form *π*-+ interaction with corresponding ligand. Each color represents a GPCR.

The pairs comprised 8 GPCRs (Figure 4) and their corresponding ligands show high ILbind score. Additional studies on agonist (or antagonist) implicate that binding pockets of these 8 GPCRs with their ligands are the same as that with agonist (or antagonist) (see details in Table S1). We analyze the binding pattern of those pairs. Result suggests that ACR prefers binding to extracellular domain of mGluR3, which is not similar to the extracellular domains of other 7 GPCRs. This may be the reason why ACR could be a high selective ligand for mGluR3. M1 mAchR, *β*_2_AR, DOR, and mGluR3 are ligand-sensitive GPCRs, which just bind to one or two kinds of ligands, whereas M3 mAchR and 5-HT receptors could bind to three or four kinds of ligand. NAI prefers to binding to extracellular domains TM2-TM3 and TM6-TM7. Residues binding to NAI prefer to form hydrogen bond in extracellular domain or TM2-TM3, while forming *π*-+ interaction in TM6-TM7. In contrast to NAI, CVD prefers binding to extracellular domain and TM4-TM5. DGX could bind to all extracellular parts of GPCR, but there is no partiality for binding patterns. For TLS, we find it mainly binds to the extracellular part of TM4-TM5 and TM6-TM7. Over 50% residues binding to TLS especially are located on the extracellular part of TM6-TM7.

## 4. Conclusions

In this paper, we manually build the AD-related pathway by consulting the KEGG pathway of AD and a mass of literature. The experimental structure determination of GPCRs still remains difficult. There are only several experimental structure fragments of the majority of GPCRs, while the comparatively complete experimental structures are rare. Therefore, the GPCR structural models used in our analysis are predicted by GPCR-I-TASSER which serves as a high-accuracy prediction tool of 3D structure of GPCRs using the experimental structures for templates. In this way, these models indirectly use the experimental structure information. The models are also aligning to the experimental structure fragments and the value of RMSD is very small, which indicates the predicted model is of great confidence. When there are complete experimental structures of GPCRs, our researching process from disease-related network to the detection of potential drug targets is able to be used to get more accurate analysis and results.

In this study, we integrate ILbind and AutoDock Vina tools to find out five potential drugs of AD, that is, ACR, CVD, DGX, NAI, and TLS. Among these, ACR is a diabetes-related drug and NAI is a nutrient of AD, while the other three drugs are designed for cardiovascular disease. These results may be helpful for the study of the off-target effect and the side effect of drugs.

## Supplementary Materials

Supplementary Materials Table S1: the first column of the table shows the GPCR family names in paper, while the second column is about the specific subtype names. The third column is the Uniprot id of each receptor. The fourth column is the selected predicted model of each receptor while the fifth column shows the model selection criterion. The sixth column of the table is agonist or antagonist of the receptor and the antagonist is marked by “(antagonist).” The seventh and eighth columns are the DrugBank id and smiles, respectively. The ninth, tenth, and eleventh columns show the second messenger, action, and effect to AD, respectively. The twelfth column is the references in paper given in PubMed id format. The thirteenth column is the binding ligands of the corresponding receptors. The fourteenth, fifteenth, and sixteenth columns are the IL-Bind score, the minimal vina affinity, and the vina affinity of the selected optimal extracellular binding site. The last column shows whether the selected optimal extracellular binding site is the same pocket with agonist or antagonist. If so, a check is marked; otherwise, a cross is marked. Table S2: dataset: 33 drugs for 22 G protein-coupled receptors in the pathway of Alzheimer's disease. Table S3: DUD-E protein-component dataset for estimating of binding score baseline. Figure S1: predicted structure model of CRHR1 and AT2R comparing with experimentally structure fragments. For CRHR1 model (left), the experimentally structure fragment 3EHU is painted in blue and located on extracellular domain marked by a blue transparent circle, while the fragment 4K5Y is painted in yellow and located on the transmembrane domain marked by a yellow transparent circle. The picture shows that the alignment of the CRHR1 model to the two fragments is good. And the RMSD of CRHR1 model aligning to 3EHU and 4K5Y is 1.16 Å and 0.66 Å, respectively, which indicates that the predicted structure model of CRHR1 is of great confidence. For AT2R model (right), there is no experimentally structured fragment. The extracellular domain marked by a blue transparent circle is predicted as coil by Psipred and SpineX, so we select the model meeting this criterion. Figure S2: the molecular binding site of predicted structure model of *β*2AR comparing with related experimental results. Left is the predicted structure model of *β*2AR binding a molecule CVD. Middle is the actual binding site of the experimentally structure fragment of *β*2AR, in which 2RH1 binds to a molecule CAU. Right about the experimentally structure of *β*1AR (4AMJ) binding to CVD, which shows an actual target of CVD. And *β*2AR is also the target of CVD.
