# Supplementary figures and images for: A Novel Method for Drug Screen to Regulate G Protein-Coupled Receptors in the Metabolic Network of Alzheimer's Disease
Source: Biomed Res Int. 2018 Feb 20;2018:5486403. doi: 10.1155/2018/5486403 (PMC5838471; doi:10.1155/2018/5486403)

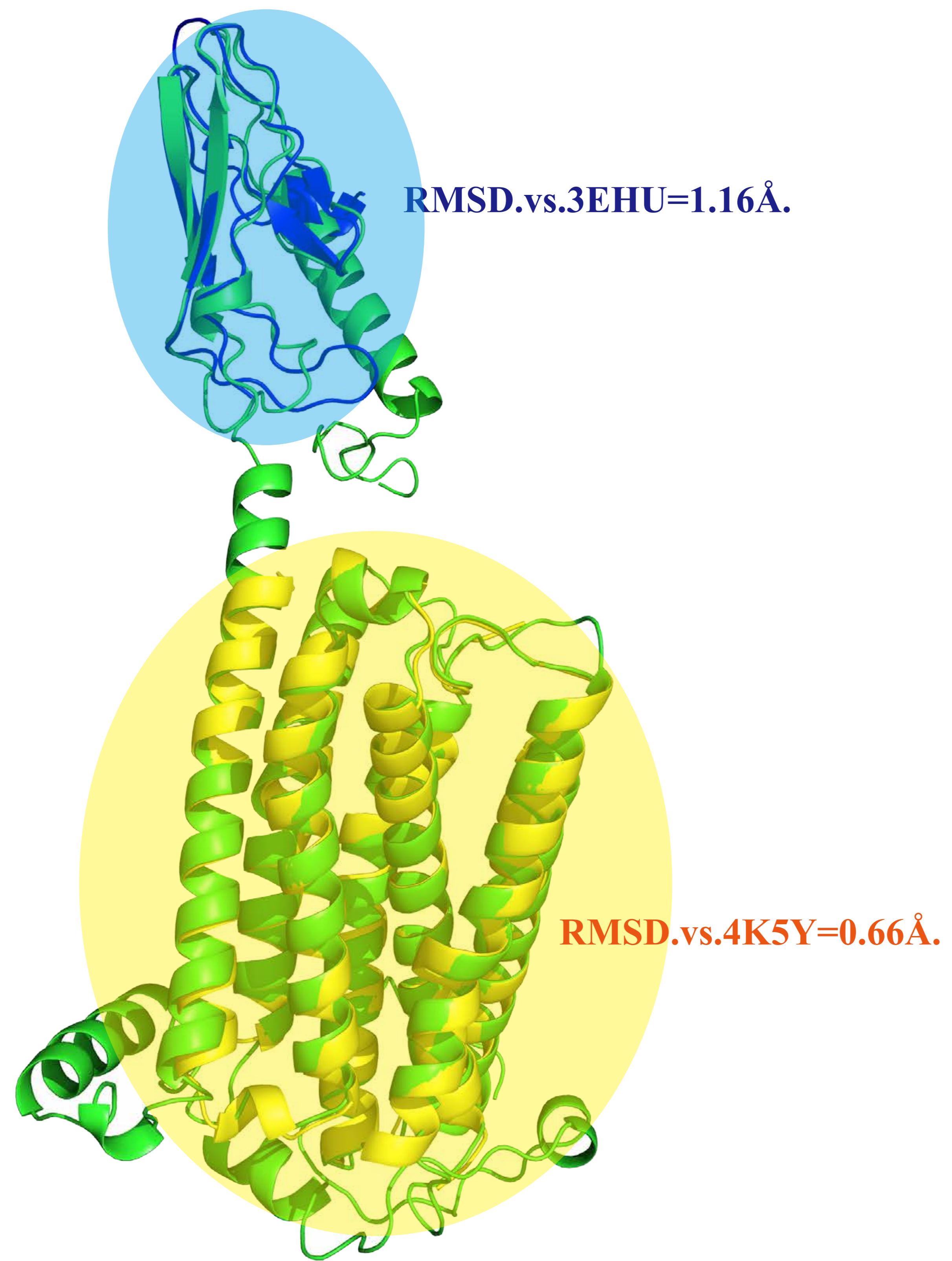

**CRHR1 Model**

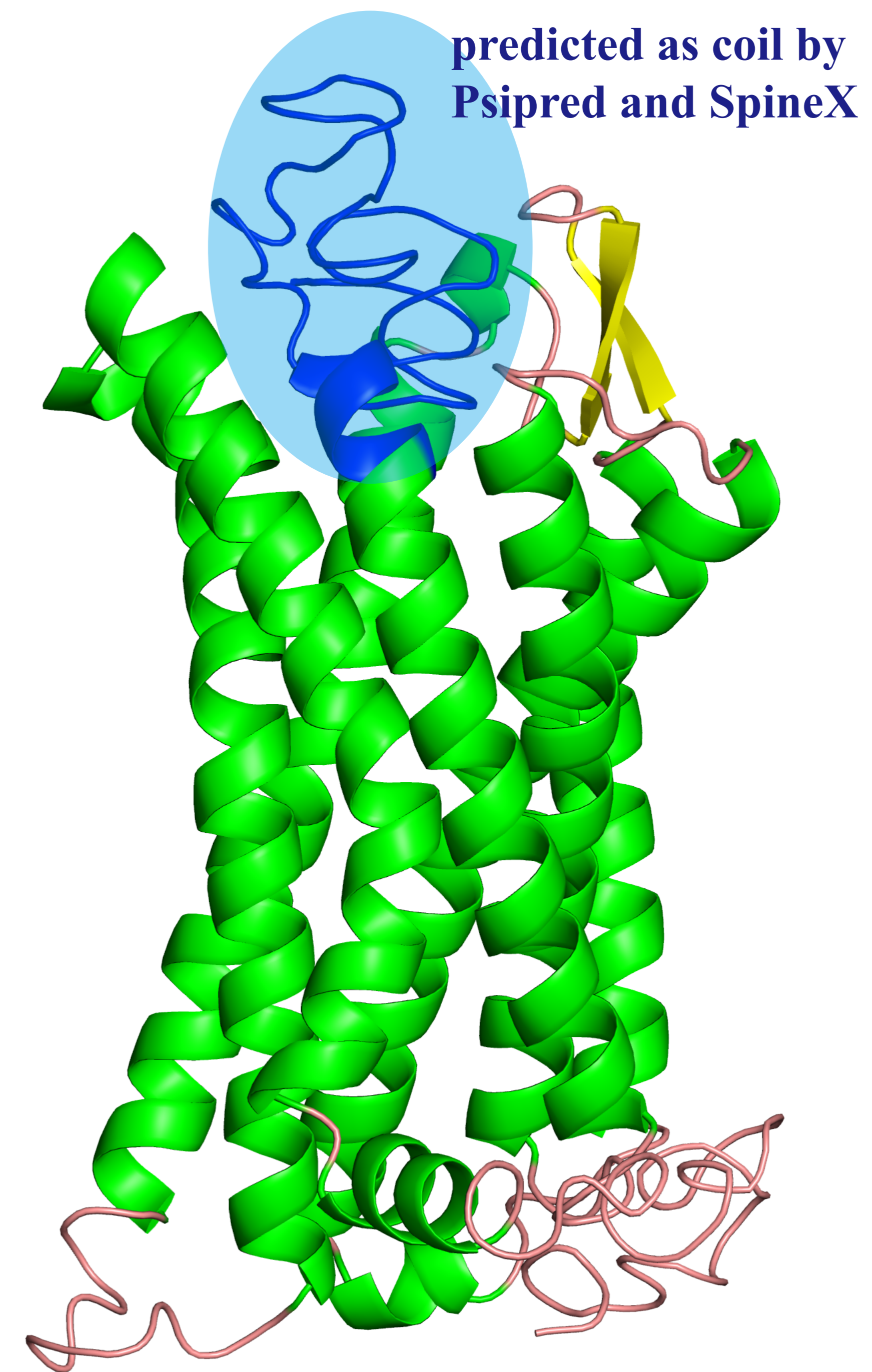

**AT2R Model**

Supplement: Supplementary Materials — Table S1: the first column of the table shows the GPCR family names in paper, while the second column is about the specific subtype names. The third column is the Uniprot id of each receptor. The fourth column is the selected predicted model of each receptor while the fifth column shows the model selection criterion. The sixth column of the table is agonist or antagonist of the receptor and the antagonist is marked by “(antagonist).” The seventh and eighth columns are the DrugBank id and smiles, respectively. The ninth, tenth, and eleventh columns show the second messenger, action, and effect to AD, respectively. The twelfth column is the references in paper given in PubMed id format. The thirteenth column is the binding ligands of the corresponding receptors. The fourteenth, fifteenth, and sixteenth columns are the IL-Bind score, the minimal vina affinity, and the vina affinity of the selected optimal extracellular binding site. The last column shows whether the selected optimal extracellular binding site is the same pocket with agonist or antagonist. If so, a check is marked; otherwise, a cross is marked. Table S2: dataset: 33 drugs for 22 G protein-coupled receptors in the pathway of Alzheimer's disease. Table S3: DUD-E protein-component dataset for estimating of binding score baseline. Figure S1: predicted structure model of CRHR1 and AT2R comparing with experimentally structure fragments. For CRHR1 model (left), the experimentally structure fragment 3EHU is painted in blue and located on extracellular domain marked by a blue transparent circle, while the fragment 4K5Y is painted in yellow and located on the transmembrane domain marked by a yellow transparent circle. The picture shows that the alignment of the CRHR1 model to the two fragments is good. And the RMSD of CRHR1 model aligning to 3EHU and 4K5Y is 1.16 Å and 0.66 Å, respectively, which indicates that the predicted structure model of CRHR1 is of great confidence. For AT2R model (right), there is no expe [file 5486403.f1.zip › Fig.S1_BMRI_2188242.pdf]

**$\beta$ 2AR model**

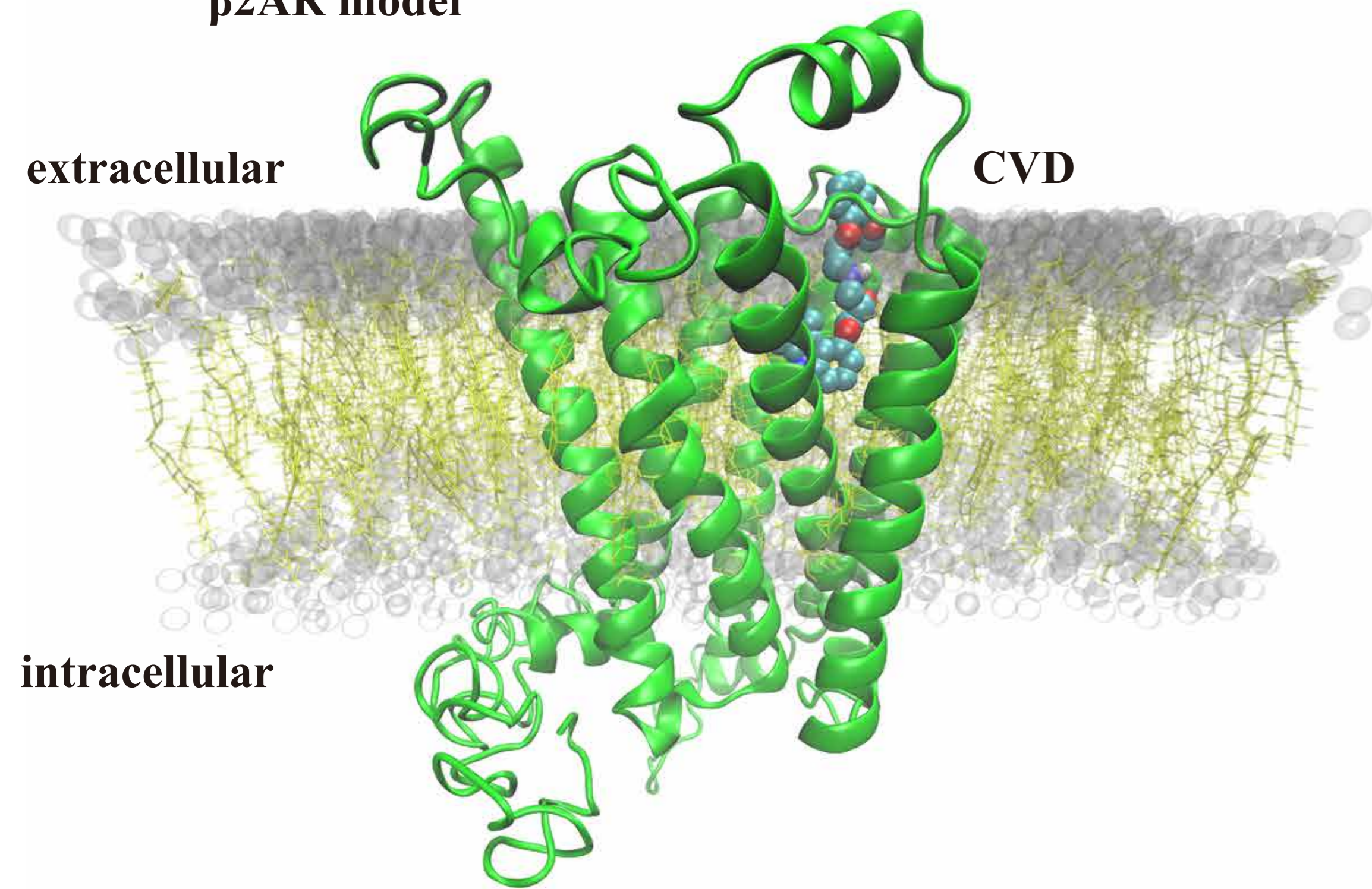

**$\beta$ 2AR 2RH1**

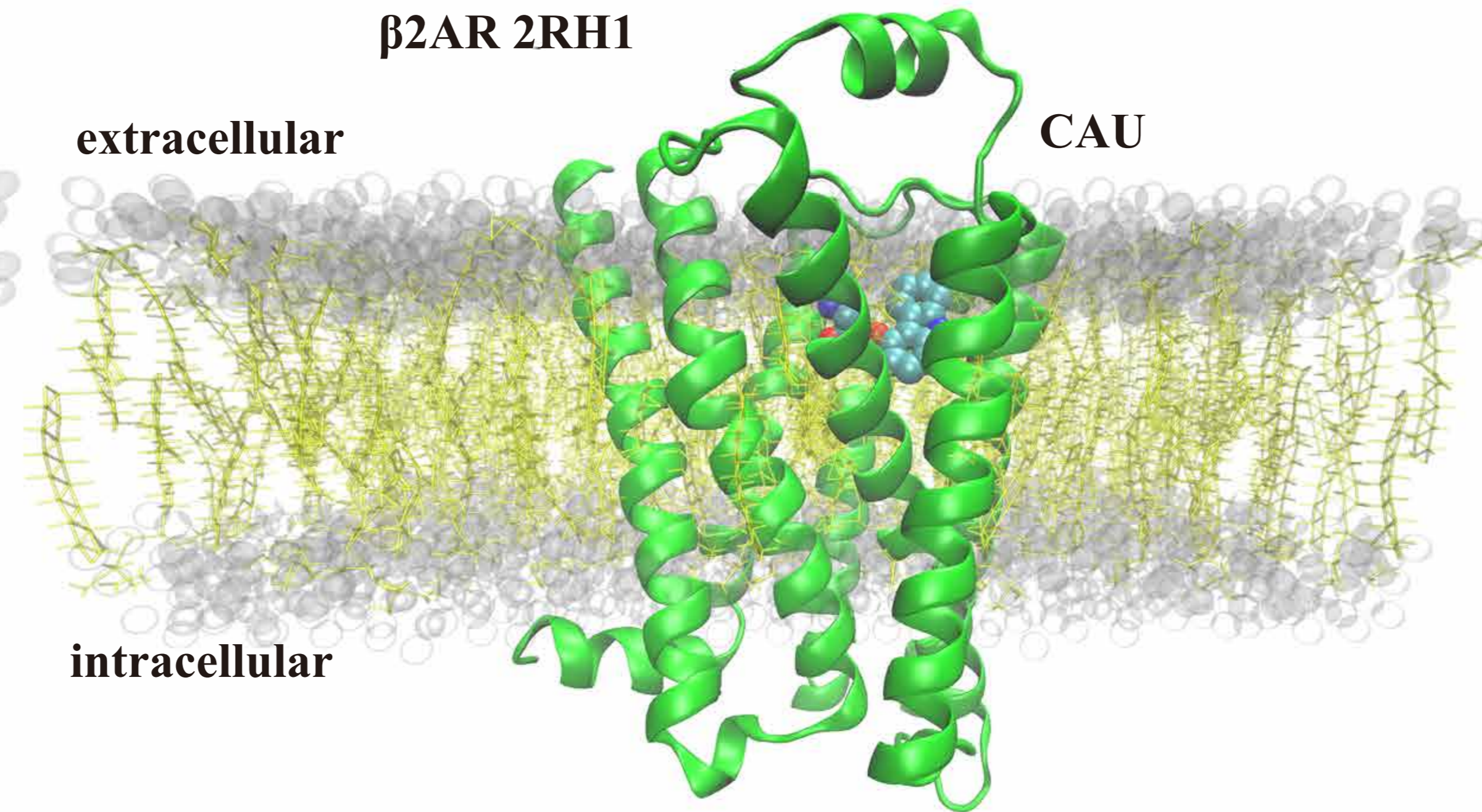

**$\beta$ 1AR 4AMJ**

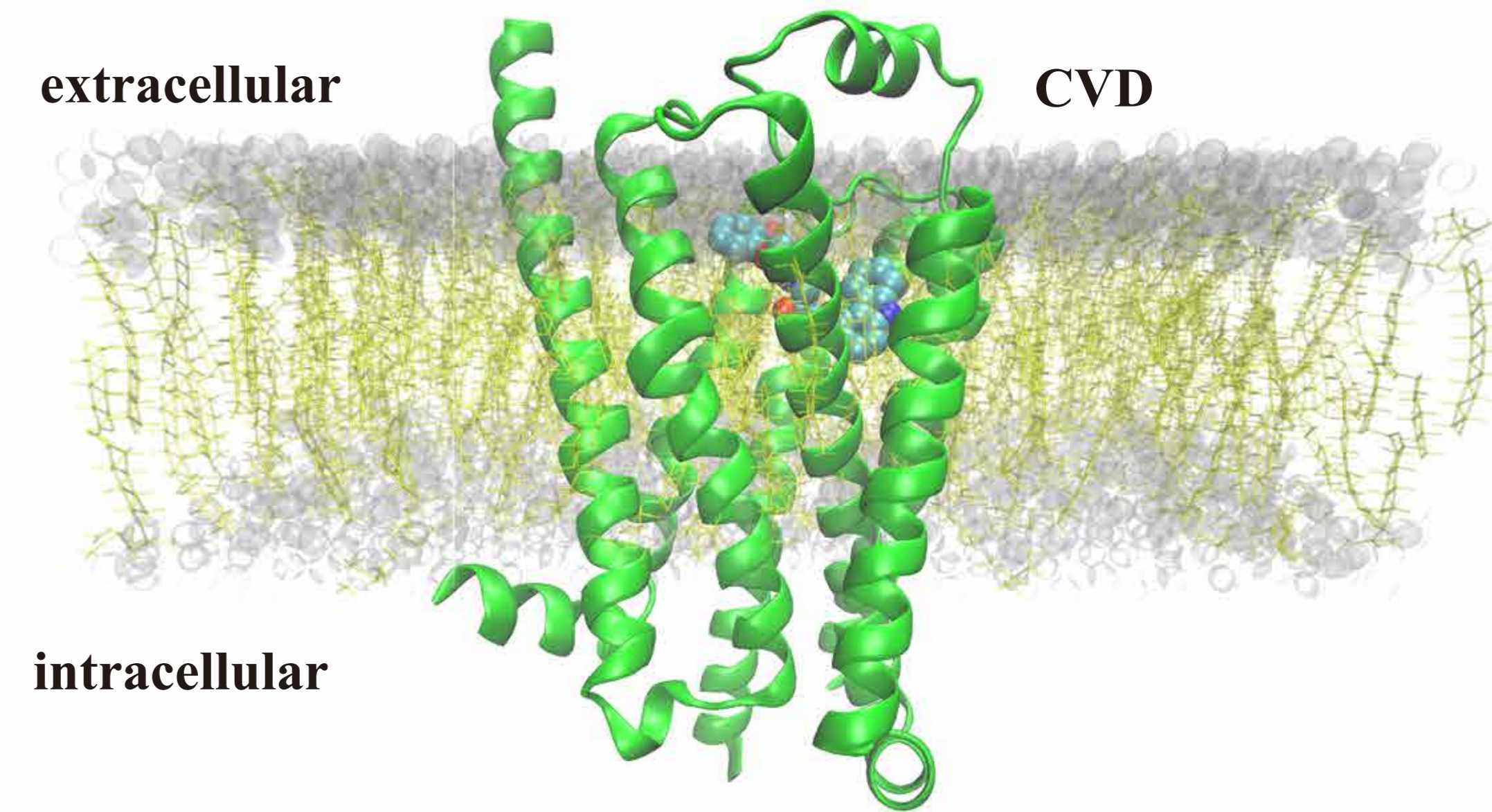

Supplement: Supplementary Materials — Table S1: the first column of the table shows the GPCR family names in paper, while the second column is about the specific subtype names. The third column is the Uniprot id of each receptor. The fourth column is the selected predicted model of each receptor while the fifth column shows the model selection criterion. The sixth column of the table is agonist or antagonist of the receptor and the antagonist is marked by “(antagonist).” The seventh and eighth columns are the DrugBank id and smiles, respectively. The ninth, tenth, and eleventh columns show the second messenger, action, and effect to AD, respectively. The twelfth column is the references in paper given in PubMed id format. The thirteenth column is the binding ligands of the corresponding receptors. The fourteenth, fifteenth, and sixteenth columns are the IL-Bind score, the minimal vina affinity, and the vina affinity of the selected optimal extracellular binding site. The last column shows whether the selected optimal extracellular binding site is the same pocket with agonist or antagonist. If so, a check is marked; otherwise, a cross is marked. Table S2: dataset: 33 drugs for 22 G protein-coupled receptors in the pathway of Alzheimer's disease. Table S3: DUD-E protein-component dataset for estimating of binding score baseline. Figure S1: predicted structure model of CRHR1 and AT2R comparing with experimentally structure fragments. For CRHR1 model (left), the experimentally structure fragment 3EHU is painted in blue and located on extracellular domain marked by a blue transparent circle, while the fragment 4K5Y is painted in yellow and located on the transmembrane domain marked by a yellow transparent circle. The picture shows that the alignment of the CRHR1 model to the two fragments is good. And the RMSD of CRHR1 model aligning to 3EHU and 4K5Y is 1.16 Å and 0.66 Å, respectively, which indicates that the predicted structure model of CRHR1 is of great confidence. For AT2R model (right), there is no expe [file 5486403.f1.zip › Fig.S2_BMRI_2188243.pdf]
